# Coming soon to a screen near you

**DOI:** 10.7554/eLife.02619

**Published:** 2014-04-29

**Authors:** Melissa Harrison

**Affiliations:** *eLife*, Cambridge, United Kingdomproduction@elifesciences.org

**Keywords:** scientific publishing, open access, eLife, xml, repositories

## Abstract

As *eLife* starts to publish the accepted versions of certain Research Articles, we explain what happens once a manuscript has been accepted for publication.

Starting this month *eLife* authors will have the option of publishing the accepted version of their article—including supplementary figures, videos and source data—on the *eLife* website within 2–3 days of acceptance; see http://elifesciences.org/upcoming. Each of these ‘accepted manuscripts’ will have a DOI and will be listed in PubMed. The final ‘version of record’ will appear a few weeks after the accepted manuscript has been published.

This move is part of our commitment to improve the publication process by reducing the length of time it takes to get new results into the open.

This move (which has already been made by a number of other publishers) is part of our commitment to improve the publication process by reducing the length of time it takes to get new results into the open. Authors have always been free to upload the latest version of any manuscript submitted to or accepted by *eLife* to their own website, or to an institutional or subject repository (such as arxiv.org or biorxiv.org): the advantages of taking the accepted manuscript route at *eLife* are that the article will immediately be more visible via, for example, PubMed, Google and email alerts from *eLife*, that the article will get a DOI sooner, and that any article-level metrics associated with the article (such as page views and downloads) will include data from both the accepted version and the final version of record. Another advantage is that we will upload the manuscript for the author.

So what actually happens between a manuscript being accepted and the version of record being published? First, the editorial production team checks the files that have been submitted to our manuscript submission system, and also all the information about the authors and the article (that is, the metadata) that the authors have input during the submission process. This often involves corresponding with authors to clarify points and requesting further information or files. If the authors agree, the decision letter (which is based on the referee reports) and author response to this letter are also prepared for publication. Meanwhile, the *eLife* features team works with the authors to produce a plain-language summary of the article. The aim of this summary—which is called an *eLife* Digest—is to make the background and central findings of the article readily understandable by a broad readership. The fact that *eLife* is an open access journal already ensures that anyone who is interested in an article can read it free of charge.

The next step is to export the article to our content processing vendor. I have deliberately avoided the term typesetter here because producing the PDF version of the article is just a small part of the whole production process today. This vendor converts the Word file submitted by the author, plus all the metadata—such as the author names and affiliations, author contributions, keywords, funding details and so on—into a XML file (see below). The figures, figure supplements, source data, videos and other subsidiary files are also resized and renamed. On completion of this process the content is pushed into an online proofing service, and authors are sent an email with a link that allows them to check their article. Authors can also download a PDF version of the proof, but all corrections have to be entered via the online proofing service.

The use of XML (short for extensible mark up language) brings many advantages, which is why it is the format of choice for most scientific publishers. First, it is straightforward to generate HTML and PDF versions of the article from the XML file; the XML file is also used to populate the Lens view of eLife articles. Second, XML allows us to deliver our content to third-party sites such as PubMed and PubMed Central. Third, the availability of the XML versions of articles supports text and data mining.

We are currently looking into other ways of using XML and other digital technologies to improve the scientific publication process. For example, we are keen to reduce the time between acceptance and publication of the final version of record. We’d also like to improve the submission process and the presentation of research articles (and their underlying data), so feedback and suggestions are welcome.

